# Flow-cytometry reveals mitochondrial DNA accumulation in *Saccharomyces cerevisiae* cells during cell cycle arrest

**DOI:** 10.3389/fcell.2024.1497652

**Published:** 2024-12-16

**Authors:** Elena Yu Potapenko, Nataliia D. Kashko, Dmitry A. Knorre

**Affiliations:** ^1^ A. N. Belozersky Institute of Physico-Chemical Biology, Lomonosov Moscow State University, Moscow, Russia; ^2^ Faculty of Bioengineering and Bioinformatics, Lomonosov Moscow State University, Moscow, Russia

**Keywords:** mtDNA, yeast, mtDNA copy number control, mtDNA copy number, cell cycle arrest, cell cycle defect

## Abstract

Mitochondria are semi-autonomous organelles containing their own DNA (mtDNA), which is replicated independently of nuclear DNA (nDNA). While cell cycle arrest halts nDNA replication, mtDNA replication continues. In *Saccharomyces cerevisiae*, flow cytometry enables semi-quantitative estimation of mtDNA levels by measuring the difference in signals between cells lacking mtDNA and those containing mtDNA. In this study, we used flow cytometry to investigate mtDNA accumulation in yeast cells under G1 and G2 phase cell cycle arrest conditions utilising thermosensitive mutants *cdc4-3* and *cdc15-2*. In line with the previous studies, cell cycle arrest induced a several-fold accumulation of mtDNA in both mutants. The total DNA levels in arrested cells correlated with cell forward scattering, suggesting a relationship between individual cell mtDNA quantity and size. In cell cycle-arrested cells, we observed no correlation between cell size and intercellular mtDNA copy number variability. This implies that as cell size increases during arrest, the mtDNA content remains within a specific limited range for each size class. This observation suggests that mtDNA quantity control mechanisms can function in cell cycle-arrested cells.

## Introduction

Mitochondrial DNA (mtDNA) is a semi-autonomous genome present in almost all eukaryotic species (Roger et al., 2017). It encodes essential components of the respiratory chain and F_O_F_1_ ATP-synthase, as well as the mitochondrial translation machinery (Lang et al., 1999). Eukaryotic cells usually harbour multiple copies of mtDNA that are replicated independently of nuclear DNA (nDNA) replication. For example, if *Saccharomyces cerevisiae* cells are arrested in G1 phase by a mating pheromone, alpha-factor, the amount of mtDNA continues increasing (Petes and Fangman, 1973). Accumulation of mtDNA in baker’s yeast cells has also been shown under conditions of cell cycle arrest in CDC (Cell Division Cycle) mutants. MtDNA continued replication upon cell cycle arrest in both G1 and G2 phases, induced by incubation of thermo-sensitive *cdc* yeast cells under non-permissive temperature (Newlon and Fangman, 1975). Replication of mtDNA has been reported in cell-cycle arrested cells of other yeast species (Sazer and Sherwood, 1990) as well as in mammalian cells (Lee et al., 2000).

Despite this semi-autonomy, multiple mechanisms ensure maintenance of mtDNA amount per cell within a certain range. This is necessary to enable mitochondrial transcription to balance the nuclear gene transcription levels (Clay Montier et al., 2009). Some mechanisms rely on partial synchronisation of nuclear and mitochondrial DNA replication. For instance, in HeLa cells, initiation of mtDNA replication happens predominantly in the G1 phase and is partially suppressed in the S phase of the cell cycle (Chatre and Ricchetti, 2013). At the same time, mtDNA deficiency can cause mitochondrial dysfunction and prevent cell cycle progression providing more time for mtDNA replication and mitochondrial biogenesis (Singh, 2004). Together, these mechanisms allow mtDNA levels to be balanced against nDNA levels by decreasing or increasing the duration of the cell cycle. Furthermore, a recent study has shown that in yeast *S.cerevisiae* the copy number of mtDNA scales with the volume of the cell, which depends on the rate of cell cycle progression. This scaling is mediated by the concentration of the proteins which limit mtDNA replication, namely, mitochondrial DNA polymerase Mip1p, and mtDNA-packaging factor Abf2p (Seel et al., 2023).

Another, though non-mutually exclusive, mechanism regulates mtDNA replication and degradation depending on metabolic cues, such as nucleotide availability. This mechanism relies on autophagy and can act independently of the cell cycle phase (Medeiros et al., 2018). This raises the question of whether mtDNA copy number per cell can be regulated in cell cycle-arrested cells, or if under conditions of cell cycle arrest, the amount of mtDNA will drift, potentially making cell populations extremely non-uniform in mtDNA copy number.

Most studies assessing mtDNA concentration rely on quantification of bulk mtDNA in the entire yeast population using quantitative PCR (Galeota-Sprung et al., 2022) or next-generation sequencing (Puddu et al., 2019). Meanwhile, microscopy-based approaches are time-consuming and hard to scale. At the same time, it was recently shown that in yeast *S.cerevisiae*, the relatively large mitochondrial genome size, as well as compact size of nDNA, enable semi-quantitative assessment of mtDNA copy number using flow cytometry with DNA-intercalating agents (Potapenko et al., 2023; Putnam, 2024).

Here, using flow cytometry, we assessed the accumulation of mtDNA in CDC mutants incubated at non-permissive temperatures, ensuring cell cycle arrest in either G1 or G2 cell cycle phase. mtDNA amount increased in arrested cells and scaled with forward scattering, which is proportional to cell size. Moreover, flow cytometry data enabled us to calculate population-based parameters, such as variability of mtDNA copy number in proliferating and arrested cells. Our data indicate that under conditions of cell cycle arrest, cells of similar size are more uniform in mtDNA copy number than might be expected. This suggests that mtDNA copy number can be maintained independently of cell cycle progression.

## Methods

### Strains and growth medium

In this study we used *W303-1A* laboratory strain (*MATa ade2-101 his3-11 trp1-1 can1-100 leu2-3*) as well as *rho* ^*0*^ derivative mutant that was obtained previously (Potapenko et al., 2023). We also utilised *cdc4-3* (Li et al., 2011) and *cdc15-2* thermosensitive strains (Zyrina et al., 2015). We depleted these strains of mtDNA by incubation of the cells in YPD supplemented with 20 µM of ethidium bromide, as described previously (Zyrina et al., 2017). We verified that these strains are incapable of growing on non-fermentable carbon sources (Supplementary Figure S1) and show no 4′,6-diamidino-2-phenylindole (DAPI) staining with the exception of the nucleus (Supplementary Figure S2).

### Cell cycle arrest

Each strain was grown overnight at 25°C in liquid YPD. We inoculated a low number of cells to ensure that by the start of the experiment, cells were in the exponential growth phase (cell density below 2 ✕ 10^6^/mL). The cell suspensions were separated into two pools and grown up to exponential growth phase overnight: one pool was immediately fixed in 70% ethanol (25°C cells, control cells), while the other underwent thermal arrest by incubating at 37°C for 6 h (37°C cells, arrested cells), followed by another optical density measurement and fixation in 70% ethanol. After each arrest, we checked the shape of the cells and the absence of buds to ensure the efficiency of the cell cycle arrest.

### DNA isolation

To quantify mtDNA/nDNA ratios and increase in mtDNA copy numbers, we used yeast cells fixed in ethanol, as described above. The cells were washed twice with deionized water (mQ) to remove ethanol, then resuspended in 300 μL of mQ. Cell walls were removed by incubating yeast cells with 100 μL of Lyticase from *Arthrobacter luteus* (Sigma-Aldrich, stock solution concentration 75 mg/mL, 20 kU/g) and 2 μL of beta-mercaptoethanol. The mixture was incubated at 37°C for 30 min with periodic manual stirring. Next, 50 μL of 10% SDS was added, and the mixture was incubated at 65°C for another 30 min with stirring. Following this, 300 μL of 9 M ammonium acetate was added, cooled at −20°C for 5 min, and incubated at 4°C for 20 min. The mixture was centrifuged at 13,000 rpm for 10 min at 4°C, and the supernatant (approximately 650 μL) was transferred to a new tube. An equal volume of chloroform was added, incubated at 4°C for 10 min, and centrifuged. The aqueous phase was collected, and 0.7 volumes of isopropanol were added. After a 10-minute incubation at 4°C, the mixture was centrifuged, and the pellet was washed twice with ethanol. The pellet was dried and dissolved in 25–40 μL of deionized water, depending on the initial cell count. To remove RNA, 0.1 μL of RNase A (10 mg/mL Fermentas, to a final concentration of 25–40 μg/mL) was added, and the mixture was incubated at 37°C for 30 min.

### qPCR

To estimate the mtDNA copy number by real-time PCR, two sets of primers to mtDNA and one to nDNA were used [(Kashko et al., 2024), see the sequences in Supplementary Table S1].

Quantitative PCR was performed on a Bio-Rad instrument using the corresponding Bio-Rad CFX Manager Software. The reaction was carried out in a volume of 25 μL. Samples with primers Mito1 and Mito2 were placed in one plate, with a common annealing temperature of 59°C (the lower of the two annealing temperatures was selected), samples with primer ACT1 were placed separately, with an annealing temperature of 55°C. The efficiency of the primers was tested in each plate in five dilutions (the top dilution had a concentration of 10 ng/μL, then down in increments of 5 or 10).

### Staining of yeast cells with PI and Sytox Green

Arrested and control yeast cells were fixed in 70% ethanol, then washed three times with sodium citrate buffer (50 mM, pH 7.0). The cells were incubated with 1.5 μL of RNase A (10 mg/mL, Fermentas) in 0.5 mL of sodium citrate buffer (final concentration 30 μg/mL) for 2 h at 37°C with stirring. Following this, 5 μL of proteinase K (20 mg/mL in 50% glycerol + TE buffer) was added, and the samples were incubated for 1 h at 50°C. After cooling, the samples were sonicated to disperse cell clumps without generating cell debris. The same fixation protocol was used for PI and Sytox Green stainings.

Finally, either 5 μL of propidium iodide (PI, 0.5 mg/mL, Sigma-Aldrich) or Sytox Green (Invitrogen) was added to achieve a final concentration of 5 μg/mL for PI or 0.625 μM for Sytox Green. Samples were wrapped in foil and incubated at 4°C for at least 1 h for PI and 30 min for Sytox Green. Treated samples were stored at 4°C for up to several weeks without significant fluorophore fading if protected from light.

### Flow cytometry data acquisition and analysis

The PI and Sytox Green stained samples were assessed on a CytoFlex (Beckman Coulter) device. For PI, the PC5.5 emission filter (wavelength 690/50 nm) was used; for SYTOX Green, the FITC emission filter (wavelength 525/40 nm) was used. In the FITC-A channel, the Sytox Green signal was three orders of magnitude higher than the autofluorescence of the cells that underwent the same fixation procedure but without the addition of Sytox Green (Supplementary Figure S3). The results were processed using the flowCore package of Bioconductor (Hahne et al., 2009). Before the analysis, the fluorescent signals were log-transformed (base = 2). The positions of 1n and 2n peaks were determined in R using the multimode package (Ameijeiras-Alonso et al., 2021) and verified manually, as described previously (Potapenko et al., 2023). For each experiment, 50,000 events were measured. The data in the text and figures is shown as average ±standard deviation calculated from at least three independent experiments. To calculate regression coefficients, we used standard tidyverse R packages (Wickham et al., 2019) and linear model approximations embedded in the geom_smooth (method = “lm”) method.

## Results

**FIGURE 1:**
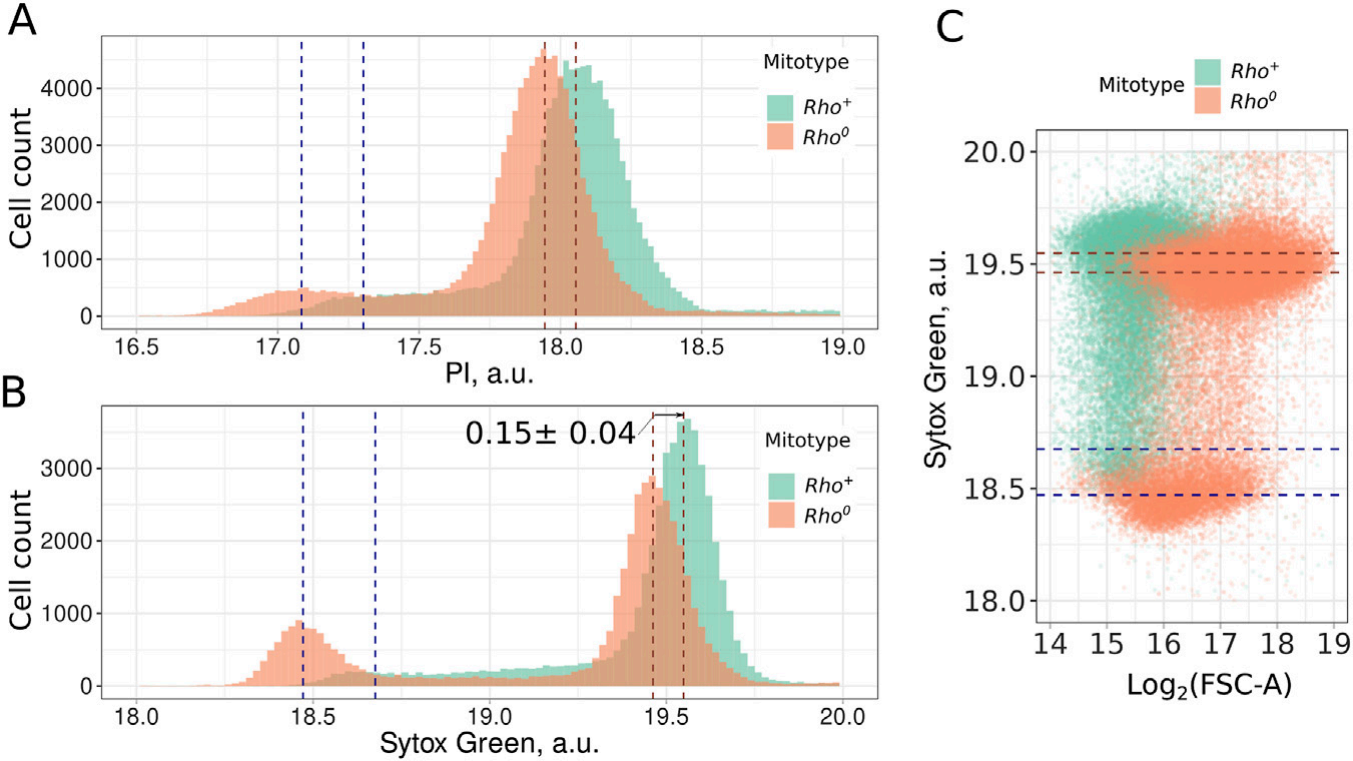
Mitochondrial DNA contributes to the Propidium Iodide **(A)** and Sytox Green **(B)** signal in yeast cells. Dotted lines indicate the position of distribution modes for 1n and 2n peaks; **(C)** Higher intensity of Sytox Green signal in *rho* ^*+*^ cells compared to *rho* ^*0*^ cells cannot be explained by the larger cell size of *rho* ^*+*^ cells. FSC-A (forward scattering area) provided a semi-quantitative estimate for the cell size.

To ensure that mtDNA signal can be detected as surplus to the nDNA signal of the cell, we compared *rho* ^*0*^ and *rho* ^*+*^ strains stained with two standard DNA-intercalating fluorescent dyes: propidium iodide (PI) and Sytox Green. For both dyes, we found that *rho* ^*0*^ cells lacking mitochondrial DNA exhibit lower fluorescence values for both 1n and 2n peaks (Figures 1A, B). Flow cytometry analysis of cells stained with DNA-intercalating agents typically reveals three distinct subpopulations: cells with single chromosomes (1n, G1-phase), cells with duplicated chromosomes (2n, G2 and M-phase), and cells undergoing DNA replication (S-phase). It should be mentioned that in the case of exponentially growing *rho* ^*+*^ cells, it is not always possible to identify the position of the 1n peak (Figures 1A, B). To check if high Sytox Green fluorescence intensity of the *rho* ^*+*^ cells can be explained by non-specific fluorescence proportional to the cell size, we compared the sizes of *rho* ^*+*^ and *rho* ^*0*^ cells. Figure 1C shows that the *rho* ^*0*^ cells have higher forward scattering, indicating that they are larger than *rho* ^*+*^ cells. Thus, the difference in fluorescent signal cannot be explained by the difference in cell size.

**FIGURE 2:**
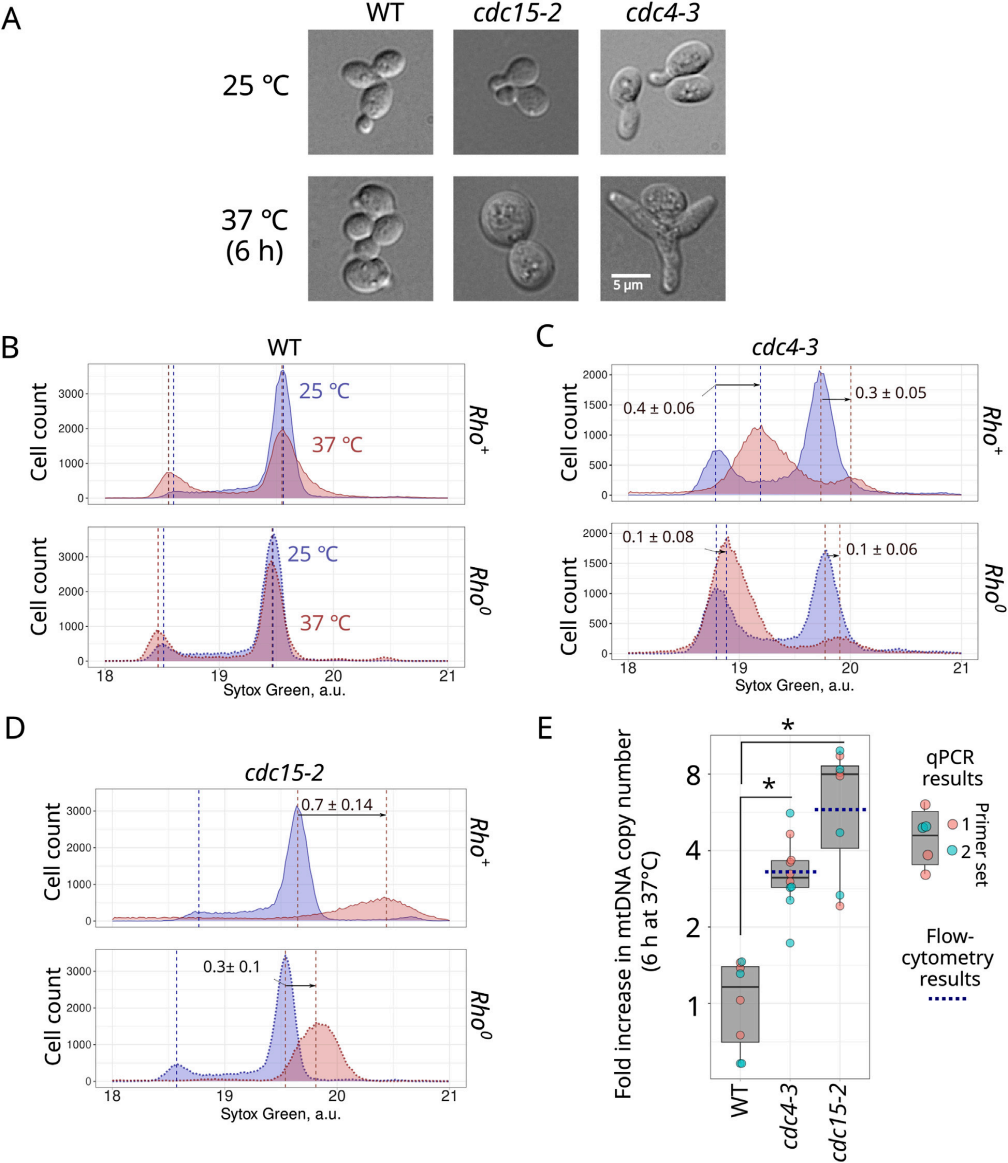
Accumulation of mtDNA during cell cycle arrest is indicated by the shift in the peaks of the Sytox Green fluorescence intensity distribution. Photograph of control cells and cell cycle arrested cells **(A)**. Sytox Green intensity distribution of *rho* ^*+*^ and *rho* ^*0*^ wild type **(B)**, *cdc4-3* **(C)**, and *cdc15-2* **(D)** incubated for 6 hours at permissive (25°C) and non-permissive (37°C) temperatures. Sytox Green intensity a.u. is calculated in log2 scale. The positions of 1n and 2n peaks were determined in R using the multimode package (see Methods). The arrows indicate shift of the peak mode as average ±standard deviation (n = 3). **(E)** Increase in mtDNA copy numbers in wild type, *cdc4-3* and *cdc15-2* strains after 6 h of incubation at 37°C assessed using qPCR with two sets of primers (see Methods). The blue dotted lines show the average increase in mtDNA calculated using flow cytometry data. * P-Value is less than 0.025 according to Wilcoxon rank sum exact test.

Given that both dyes provided similar results, in subsequent experiments we have chosen only one of them, Sytox Green. To test whether mtDNA molecules accumulate under conditions of cell cycle arrest, we used two CDC mutants available in our laboratory: *cdc15-2* and *cdc4-3*. After 6 hours of incubation at non-permissive temperature (37°C), cells of these strains significantly increased in size (Figure 2A; Supplementary Figure S4). We detected no budded cells in these 37°C suspensions. In both *rho* ^*+*^ and *rho* ^*0*^ wild-type strains, 6 hours of incubation at the elevated temperature did not change the distribution modes of the Sytox Green signal, though it could be mentioned that the elevated temperature increased the proportion of 1n cells (Figure 2B). As expected, CDC mutant cell suspensions exhibited an increase in the proportions of either 1n cells (*cdc4-3*) or 2n cells (*cdc15-2* strain) on non-permissive temperatures. Meanwhile, cell cycle arrest shifted the position of the Sytox Green signal distribution mode to the right, suggesting an increase in total DNA content in both tested *cdc* strains (Figures 2C, D). Importantly, this increase was much more pronounced in *rho* ^*+*^ cells compared to the *rho* ^*0*^ cells (Figures 2C, D), though it should be noted that *rho* ^*0*^ cells also exhibited a moderate increase in Sytox Green signal intensity; we discuss possible reasons for this increase below.

The shifts in 1n and 2n peak mode positions in *cdc15-2* and *cdc4-3* cells provided a semi-quantitative estimate of mtDNA copy number. To analyse Sytox Green intensity in individual cells, we transformed the raw flow cytometry data by taking the logarithm base two. Consequently, the position of the 1n and 2n peak modes differed by one unit (see Figures 1, 2). Accordingly, mtDNA copy number can be calculated using the shift between the *rho* ^*0*^ and *rho* ^*+*^ distribution modes of corresponding peaks (see Supplementary Figure S5 and Supplementary Table S2 for the formulas).

On average, the position of *rho* ^*+*^ cells’ 2n peak mode was 0.15 ± 0.03 greater (on a log2-transformed scale) than the position of the *rho* ^*0*^ cells’ distribution peak (Figure 1B). Assuming the size of *S.cerevisiae* mtDNA as 0.085 Mb (Foury et al., 1998), yeast 2n nDNA as 24.2 Mb (Peter et al., 2018), and no other factors significantly contribute to the Sytox Green intensity, we calculated the mtDNA copy number as 30.8 ± 6.2 copies in 2n cells (see Supplementary Figure S5 and Supplementary Table S2). This value is likely a slight overestimate because yeast cells can contain up to 60 molecules of a 2µ plasmid, each 6.3 kb in size (Chan et al., 2013). However, these plasmids in total would contribute only 378 kb, which is equivalent to 4.45 copies of mtDNA. Figure 2 shows an increase in mtDNA copy number in *cdc4-3* arrested cells by a factor of 0.4 (and 0.1 in *rho* ^*0*^ cells), suggesting a surplus of ∼36 ± 6 copies of mtDNA molecules during 6 hours of cell cycle arrest. This represents a ∼3.3 increase in the copy number of mtDNA molecules in *cdc4-3* cells. Similarly, *cdc15-2* cells arrested in G2/M phase exhibited an increase of the 2n mode value by 0.7 (and 0.3 in *rho* ^*0*^ cells) units, equivalent to ∼147 ± 24 mtDNA molecules. This represents a ∼5.8 increase in the copy number of mtDNA molecules in *cdc15-2* cells.

To verify these assessments with an independent method, we quantified the increase in mtDNA copy number using qPCR. We selected primer sets for two different regions of mtDNA and the *ACT1* gene in nDNA (Kashko et al., 2024) Although day-to-day qPCR results showed high variability of mtDNA to *ACT1* numbers (Supplementary Figure S6), this enabled us to calculate the increase in mtDNA copy numbers in *cdc4-3* and *cdc15-2* arrested cells (Figure 2E). On average, the observed increase was 3.4 ± 1.1 fold in *cdc4-3* cells and 6.7 ± 3.0 fold in *cdc15-2* cells. These results are in a good agreement with the results obtained using flow-cytometry (Figure 2E).

**FIGURE 3:**
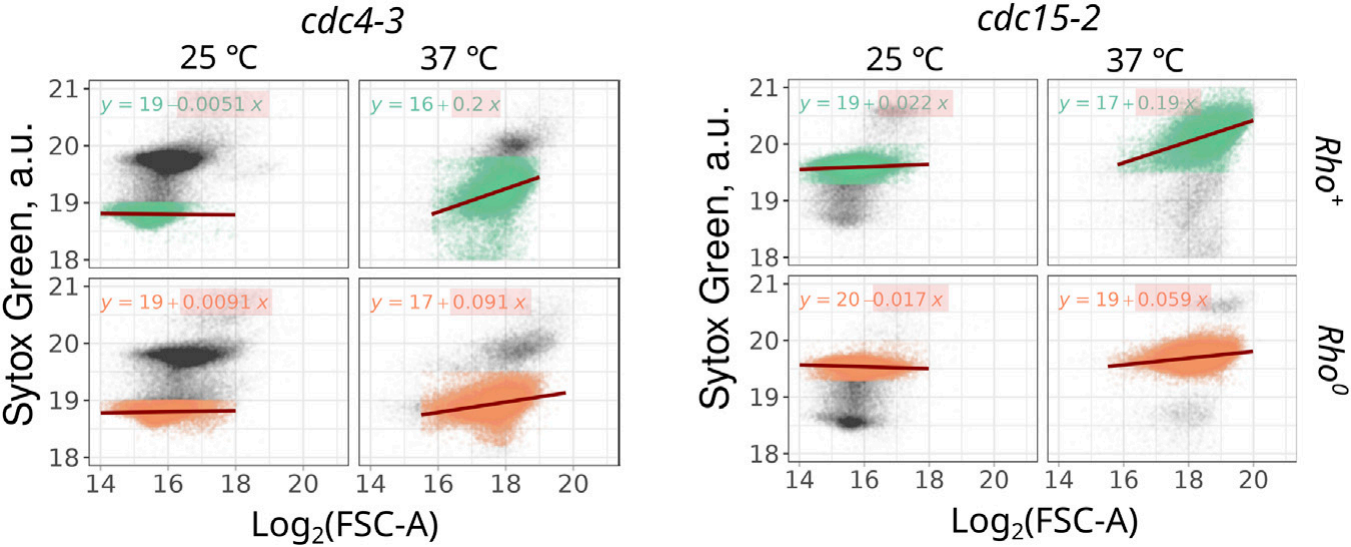
The highest correlation between the Sytox Green signal and cell size (log_2_ of forward scattering area) is observed in cell cycle-arrested *rho* ^*+*^ cell suspensions. Correlations were calculated for 1n cells in the *cdc4-3* strain (left panel) and 2n cells in the *cdc15-2* strain (right panel).

The observed increase in mtDNA copy number can be correlated with the increase of cell size or may occur independently. Flow cytometry routinely provides not only population average parameters but also estimates of individual cell sizes through measurements of forward and side light scattering. Consequently, we were able to investigate whether the Sytox Green signal and mtDNA-explained component of the signal correlate with the cell size. We examined the correlation between Sytox Green intensity and forward scattering (FSC-A) in thermo-sensitive mutants. For this analysis, we focused on events corresponding to 1n cells in the *cdc4-3* strain and 2n cells in the *cdc15-2* strain. Linear regression analysis between these two parameters revealed that in *cdc*-mutant cells proliferating at permissive temperature (25°C), Sytox Green signal is virtually independent of cell size (Figure 3). However, during cell cycle arrest, *rho* ^*+*^ cells exhibit a strong dependency between Sytox Green signal (which we believe depends mainly on mtDNA amount) and FSC-A (Figure 3). The regression coefficients of Sytox Green intensity dependence on size (FSC-A) for *rho* ^*+*^ cells were at least twice as large as those for *rho* ^*0*^ cells (Figure 3).

**FIGURE 4:**
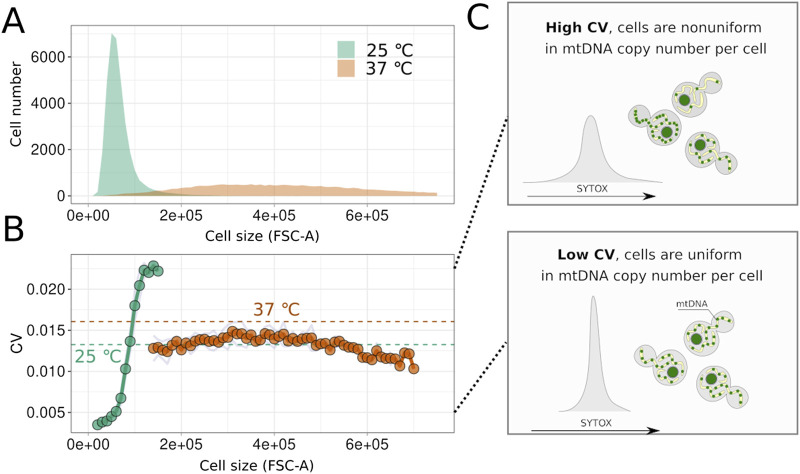
Yeast cells remain homogeneous in mtDNA content despite the increase in size during cell cycle arrest. **(A)** Distributions of FSC-A values for cells of strain *cdc15-2* before and after incubation at the non-permissive temperature. **(B)** Values of the variation coefficient calculated for arrested and non-arrested cells in different size bins (average values, n = 3). Dotted line shows population average without binning on cell size. **(C)** Schematic illustrating that the variation coefficient reflects the heterogeneity of cells in the Sytox Green signal, which in turn depends on the mtDNA copy number in the arrested cells.

Next, we aimed to determine whether mtDNA amount is regulated during cell cycle arrest or if the number of mtDNA molecules increases stochastically. We hypothesised that stochastic increases would result in heterogeneity in mtDNA content and, consequently, in the Sytox Green signal within the cell suspension. Flow cytometry allows assessment of cell suspension homogeneity in terms of Sytox Green signal and, therefore, mtDNA content. This comparison is feasible only for arrested cells with a constant amount of nuclear DNA (nDNA). To exclude potential variability in Sytox Green signal from nDNA of cells at different cell cycle stages, we analysed the right part of the 2n peak for both arrested and non-arrested cells of the *cdc15-2* strain, as previously described (Potapenko et al., 2023). Figure 4A demonstrates that cell cycle arrest, induced by incubating cells at a non-permissive temperature, leads to an increase in FSC-A. To exclude the impact of cell size variation, we calculated the coefficient of variation (CV) of the Sytox Green signal for yeast cells within narrow cell size bins, considering only bins containing at least 200 cells. Surprisingly, as shown in Figure 4B, CV values in *cdc15-2* G2/M-arrested cells did not increase with cell size. Moreover, comparing arrested and non-arrested cells within the same size bins revealed that arrested cells are more uniform in Sytox Green signal and, possibly, in mtDNA copy numbers (see illustration in Figure 4C). Together, this indicates that the variability of SytoxGreen signal in *cdc15-2* arrested cells is mainly explained by variation of cell size, while mtDNA copy numbers in individual cells are likely controlled in cell cycle arrested cells.

## Discussion

Our study demonstrates mtDNA accumulation during cell cycle arrest using a novel approach that estimates mtDNA quantity in individual cells. We estimate 30.8 ± 6.2 copies of mtDNA in G2/M cells, which aligns well with other qPCR-based studies reporting 20–40 molecules per haploid genome (Göke et al., 2020; Galeota-Sprung et al., 2022). It should be mentioned that the cells in the exponential growth phase show lower mtDNA content and the highest estimate of 40 molecules were made for stationary phase cells (Galeota-Sprung et al., 2022). Another study estimated 17 mtDNA copies per haploid genome in yeast cells grown in YPD, and 32 copies when grown on glycerol, a non-fermentable carbon source that induces mitochondrial biogenesis (Schrott and Osman, 2023). Importantly, the actual average number of mtDNA molecules in yeast cells is likely higher than the ratio indicated by qPCR, as a significant portion of cells contain two copies of nDNA (Figure 1). This underestimation varies depending on the proportion of cells in different cell cycle phases. An alternative approach based on next-generation sequencing, which involves mapping reads to nDNA and mtDNA and comparing coverage depths, provides slightly lower estimates of 9–30 mtDNA copies per haploid cell in exponentially growing cultures (Puddu et al., 2019; Galeota-Sprung et al., 2022).

Both qPCR and NGS methods for estimating mtDNA copy number have potential limitations. Firstly, these approaches require total DNA isolation, but the efficiency of mtDNA and nDNA extraction can vary significantly and is sensitive to the isolation method used. Secondly, both techniques rely on DNA amplification, which can occur with varying efficiency depending on local nucleotide composition and DNA integrity. This is particularly relevant fo*r S. cerevisiae* and related species, whose mitochondrial genomes are extremely AT-rich [typically over 80% AT content; (Wolters et al., 2023)], with only a small portion having GC content similar to nDNA. Consequently, it is advantageous to confirm mtDNA copy number estimates using a fundamentally different method that circumvents these limitations.

The flow cytometry-based approach also has its limitations and potential biases. Firstly, some DNA-intercalating agents, such as DAPI, can be strongly influenced by the GC-content of DNA (Kapuscinski, 1995), making it challenging to compare the contributions of mtDNA and nDNA to the total fluorescence signal. That limits the available toolkit of DNA-intercalating agents. Secondly, other factors can increase total DNA variance, including plasmids, rDNA rings, and telomeres. While it's unlikely that all of these contribute significantly to the total fluorescence signal under normal conditions [see discussion in (Potapenko et al., 2023)], we cannot completely rule out the possibility of a sharp increase in one of these factors under the cell cycle arrest conditions, as observed with rDNA in aging yeast cells (Sinclair and Guarente, 1997). The increase in Sytox Green signal in arrested *rho* ^*0*^ cells (Figures 2, 3) is likely due to one of these unidentified factors. Previous studies have shown that enlarged cells exhibit a high coefficient of variation (CV) in the fluorescence of DNA-intercalating dyes, although this effect was more pronounced in PI-stained cells and less significant with Sytox Green (Haase and Reed, 2002). To mitigate the influence of these factors, we subtracted the signal obtained from *rho* ^*0*^ cells when calculating the Sytox Green fluorescence surplus, wherever possible.

In contrast to other methods, flow cytometry produces additional data that cannot be obtained from the pooled population of cells using qPCR or NGS. For example, flow cytometry-based results provide not only an average estimate of mtDNA content in yeast cells but also an assessment of the suspension’s homogeneity for this parameter. We previously reported that *rho* ^*+*^ and *rho* ^*−*^ cells show higher variation in Sytox Green signal than *rho* ^*0*^ cells suggesting that mtDNA copy number significantly contributes to Sytox Green signal population variability (Potapenko et al., 2023). Therefore, we attribute this variation in the DNA intercalating agent signal primarily to variations in mtDNA content.

Another advantage of the flow cytometry-based approach to quantify DNA is that it allows for the evaluation of mtDNA quantity across different cell cycle phases, even in asynchronous populations. Exploiting both advantages of the method, we demonstrated that upon cell cycle arrest, the width of the Sytox Green peak notably increases, indicating greater heterogeneity in DNA content (Figure 2). However, Figure 4 reveals that this heterogeneity is mainly due to differences in cell size. It should be mentioned that this conclusion relies on the assumption that FSC-A is correlated with cell size while the other properties of yeast cells, such as the shape of the cells, might also contribute to light scattering. Nonetheless, within groups of similarly sized cells (analysed using a narrow sliding window), the coefficient of variation remains relatively low, not exceeding that of large cells before cell cycle arrest. This result suggests that intercellular variability of mtDNA copy number is still within certain boundaries in cell cycle-arrested cells for each size class.

In conclusion, our study provides a novel, individual cell-level estimate of mtDNA copy number in both proliferating and cell cycle-arrested yeast using an unconventional flow cytometry-based approach. The ability of eukaryotic cells to maintain mtDNA copy number within physiological thresholds is crucial for their functioning. In humans, disruption of this regulation leads to mitochondrial disorders, such as mtDNA depletion syndrome (Filograna et al., 2021). Our findings confirm the ability of the mitochondrial genome to be replicated independently of nDNA in both G1 and G2/M phases of cell cycle. Importantly, we show that mtDNA quantity in yeast cells remains regulated even under cell cycle arrest conditions. Furthermore, our data implies that cell growth, even to substantial sizes, does not disrupt this regulatory mechanism. Our results support the notion that mtDNA copy number regulation remains active in post-mitotic cells that have ceased nDNA replication.

## Data Availability

The datasets presented in this study can be found in online repositories. The names of the repository/repositories and accession number(s) can be found below: https://doi.org/10.5281/zenodo.14018551.

## References

[B1] Ameijeiras-Alonso J. Crujeiras R. M. Rodriguez-Casal A. (2021). Multimode: an R package for mode assessment. J. Stat. Softw. 97, 1–32. 10.18637/jss.v097.i09

[B2] Chan K.-M. Liu Y.-T. Ma C.-H. Jayaram M. Sau S. (2013). The 2 micron plasmid of *Saccharomyces cerevisiae*: a miniaturized selfish genome with optimized functional competence. Plasmid 70, 2–17. 10.1016/j.plasmid.2013.03.001 23541845

[B3] Chatre L. Ricchetti M. (2013). Prevalent coordination of mitochondrial DNA transcription and initiation of replication with the cell cycle. Nucleic Acids Res. 41, 3068–3078. 10.1093/nar/gkt015 23345615 PMC3597681

[B4] Clay Montier L. L. Deng J. J. Bai Y. (2009). Number matters: control of mammalian mitochondrial DNA copy number. J. Genet. Genomics 36, 125–131. 10.1016/S1673-8527(08)60099-5 19302968 PMC4706993

[B5] Filograna R. Mennuni M. Alsina D. Larsson N.-G. (2021). Mitochondrial DNA copy number in human disease: the more the better? FEBS Lett. 595, 976–1002. 10.1002/1873-3468.14021 33314045 PMC8247411

[B6] Foury F. Roganti T. Lecrenier N. Purnelle B. (1998). The complete sequence of the mitochondrial genome of *Saccharomyces cerevisiae* . FEBS Lett. 440, 325–331. 10.1016/s0014-5793(98)01467-7 9872396

[B7] Galeota-Sprung B. Fernandez A. Sniegowski P. (2022). Changes to the mtDNA copy number during yeast culture growth. R. Soc. Open Sci. 9, 211842. 10.1098/rsos.211842 35814911 PMC9257595

[B8] Göke A. Schrott S. Mizrak A. Belyy V. Osman C. Walter P. (2020). Mrx6 regulates mitochondrial DNA copy number in *Saccharomyces cerevisiae* by engaging the evolutionarily conserved Lon protease Pim1. Mol. Biol. Cell. 31, 527–545. 10.1091/mbc.E19-08-0470 31532710 PMC7202074

[B9] Haase S. B. Reed S. I. (2002). Improved flow cytometric analysis of the budding yeast cell cycle. Cell. Cycle 1, 117–121. 10.4161/cc.1.2.114 12429922

[B10] Hahne F. LeMeur N. Brinkman R. R. Ellis B. Haaland P. Sarkar D. (2009). flowCore: a Bioconductor package for high throughput flow cytometry. BMC Bioinforma. 10, 106. 10.1186/1471-2105-10-106 PMC2684747 19358741

[B11] Kapuscinski J. (1995). DAPI: a DNA-specific fluorescent probe. Biotech. Histochem. 70, 220–233. 10.3109/10520299509108199 8580206

[B12] Kashko N. D. Karavaeva I. Glagoleva E. S. Logacheva M. D. Garushyants S. K. Knorre D. A. (2024). Inheritance bias of deletion-harbouring mtDNA in yeast: the role of copy number and intracellular selection. bioRxiv. 10.1101/2024.09.11.612442 PMC12186888 40554585

[B13] Lang B. F. Gray M. W. Burger G. (1999). Mitochondrial genome evolution and the origin of eukaryotes. Annu. Rev. Genet. 33, 351–397. 10.1146/annurev.genet.33.1.351 10690412

[B14] Lee H. C. Yin P. H. Lu C. Y. Chi C. W. Wei Y. H. (2000). Increase of mitochondria and mitochondrial DNA in response to oxidative stress in human cells. Biochem. J. 348 (Pt 2), 425–432. 10.1042/bj3480425 10816438 PMC1221082

[B15] Li Z. Vizeacoumar F. J. Bahr S. Li J. Warringer J. Vizeacoumar F. S. (2011). Systematic exploration of essential yeast gene function with temperature-sensitive mutants. Nat. Biotechnol. 29, 361–367. 10.1038/nbt.1832 21441928 PMC3286520

[B16] Medeiros T. C. Thomas R. L. Ghillebert R. Graef M. (2018). Autophagy balances mtDNA synthesis and degradation by DNA polymerase POLG during starvation. J. Cell. Biol. 217, 1601–1611. 10.1083/jcb.201801168 29519802 PMC5940314

[B17] Newlon C. S. Fangman W. L. (1975). Mitochondrial DNA synthesis in cell cycle mutants of *Saccharomyces cerevisiae* . Cell. 5, 423–428. 10.1016/0092-8674(75)90061-6 1098780

[B18] Peter J. De Chiara M. Friedrich A. Yue J.-X. Pflieger D. Bergström A. (2018). Genome evolution across 1,011 *Saccharomyces cerevisiae* isolates. Nature 556, 339–344. 10.1038/s41586-018-0030-5 29643504 PMC6784862

[B19] Petes T. D. Fangman W. L. (1973). Preferential synthesis of yeast mitochondrial DNA in alpha factor-arrested cells. Biochem. Biophys. Res. Commun. 55, 603–609. 10.1016/0006-291x(73)91186-8 4586613

[B20] Potapenko E. Y. Kashko N. D. Knorre D. A. (2023). Spontaneous mutations in *Saccharomyces cerevisiae* mtDNA increase cell-to-cell variation in mtDNA amount. Int. J. Mol. Sci. 24, 17413. 10.3390/ijms242417413 38139242 PMC10743915

[B21] Puddu F. Herzog M. Selivanova A. Wang S. Zhu J. Klein-Lavi S. (2019). Genome architecture and stability in the *Saccharomyces cerevisiae* knockout collection. Nature 573, 416–420. 10.1038/s41586-019-1549-9 31511699 PMC6774800

[B22] Putnam C. D. (2024). Loss of mitochondrial DNA is associated with reduced DNA content variability in *Saccharomyces cerevisiae* . Micropubl. Biol. 2024. 10.17912/micropub.biology.001117 PMC10964099 38533353

[B23] Roger A. J. Muñoz-Gómez S. A. Kamikawa R. (2017). The origin and diversification of mitochondria. Curr. Biol. 27, R1177–R1192. 10.1016/j.cub.2017.09.015 29112874

[B24] Sazer S. Sherwood S. W. (1990). Mitochondrial growth and DNA synthesis occur in the absence of nuclear DNA replication in fission yeast. J. Cell. Sci. 97 (Pt 3), 509–516. 10.1242/jcs.97.3.509 2074269

[B25] Schrott S. Osman C. (2023). Two mitochondrial HMG-box proteins, Cim1 and Abf2, antagonistically regulate mtDNA copy number in *Saccharomyces cerevisiae* . Nucleic Acids Res. 51, 11813–11835. 10.1093/nar/gkad849 37850632 PMC10681731

[B26] Seel A. Padovani F. Mayer M. Finster A. Bureik D. Thoma F. (2023). Regulation with cell size ensures mitochondrial DNA homeostasis during cell growth. Nat. Struct. Mol. Biol. 30, 1549–1560. 10.1038/s41594-023-01091-8 37679564 PMC10584693

[B27] Sinclair D. A. Guarente L. (1997). Extrachromosomal rDNA circles— a cause of aging in yeast. Cell. 91, 1033–1042. 10.1016/s0092-8674(00)80493-6 9428525

[B28] Singh K. K. (2004). Mitochondria damage checkpoint in apoptosis and genome stability. FEMS Yeast Res. 5, 127–132. 10.1016/j.femsyr.2004.04.008 15489195

[B29] Wickham H. Averick M. Bryan J. Chang W. McGowan L. François R. (2019). Welcome to the tidyverse. J. Open Source Softw. 4, 1686. 10.21105/joss.01686

[B30] Wolters J. F. LaBella A. L. Opulente D. A. Rokas A. Hittinger C. T. (2023). Mitochondrial genome diversity across the subphylum Saccharomycotina. Front. Microbiol. 14, 1268944. 10.3389/fmicb.2023.1268944 38075892 PMC10701893

[B31] Zyrina A. N. Smirnova E. A. Markova O. V. Severin F. F. Knorre D. A. (2017). Mitochondrial superoxide dismutase and Yap1p act as a signaling module contributing to ethanol tolerance of the yeast *Saccharomyces cerevisiae* . Appl. Environ. Microbiol. 83, e02759. 10.1128/AEM.02759-16 27864171 PMC5244310

[B32] Zyrina A. N. Sorokin M. I. Sokolov S. S. Knorre D. A. Severin F. F. (2015). Mitochondrial retrograde signaling inhibits the survival during prolong S/G2 arrest in *Saccharomyces cerevisiae* . Oncotarget 6, 44084–44094. 10.18632/oncotarget.6406 26624981 PMC4792543

