# Supplementary material for: Flow-cytometry reveals mitochondrial DNA accumulation in Saccharomyces cerevisiae cells during cell cycle arrest
Source: Front Cell Dev Biol. 2024 Dec 16;12:1497652. doi: 10.3389/fcell.2024.1497652 (PMC11683134; doi:10.3389/fcell.2024.1497652)
Supplement: Supplementary file 2 [file DataSheet1.pdf]

Supplementary materials: Flow-cytometry reveals mitochondrial DNA accumulation in yeast cells during cell cycle arrest

Elena Yu. Potapenko[1], Nataliia D. Kashko[2], Dmitry A. Knorre[1]

[1]A.N. Belozersky Institute of Physico-Chemical Biology, Lomonosov Moscow State University, 119234, Moscow, Leninskiye Gory, 1-40

[2]Faculty of Bioengineering and Bioinformatics, Lomonosov Moscow State University, 119234, Moscow, Leninskiye Gory, 1-73

* knorre@belozersky.msu.ru

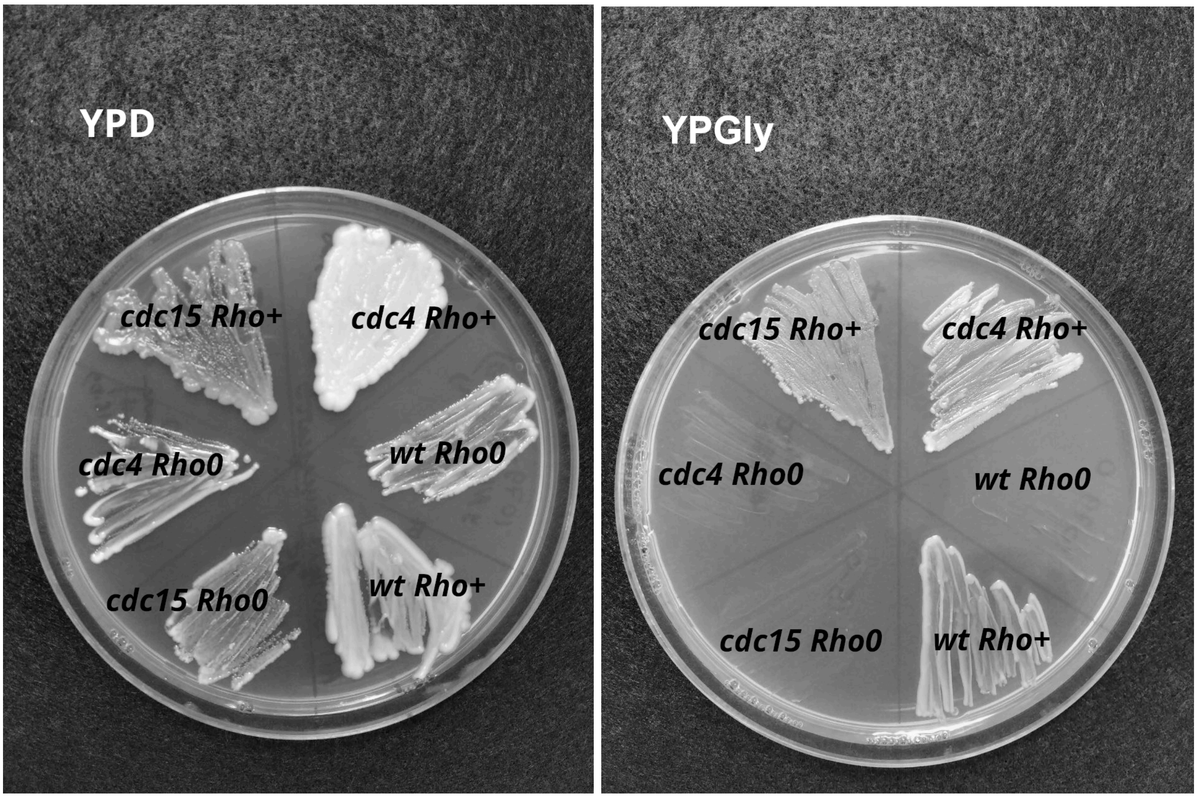

Figure S1. mtDNA-deficient *rho⁰* strains are unable to grow on media with a non-fermentable carbon source, YPGly.

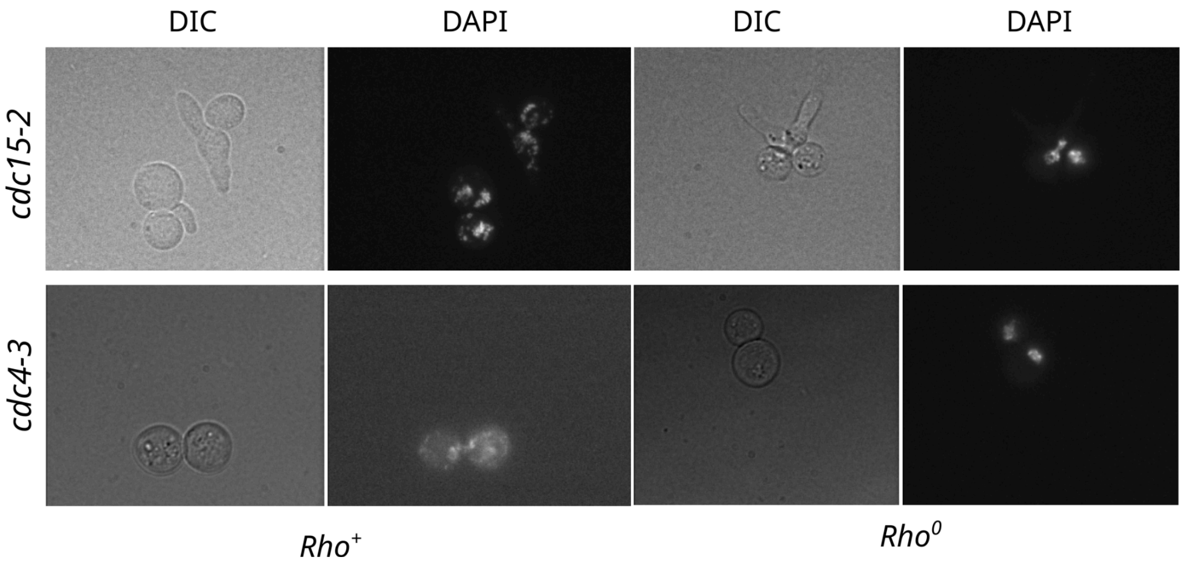

Figure S2. mtDNA-deficient *rho*^{0} *cdc15-2* and *cdc4-3* strains show no cytoplasmic DAPI staining, proving the absence of mtDNA.

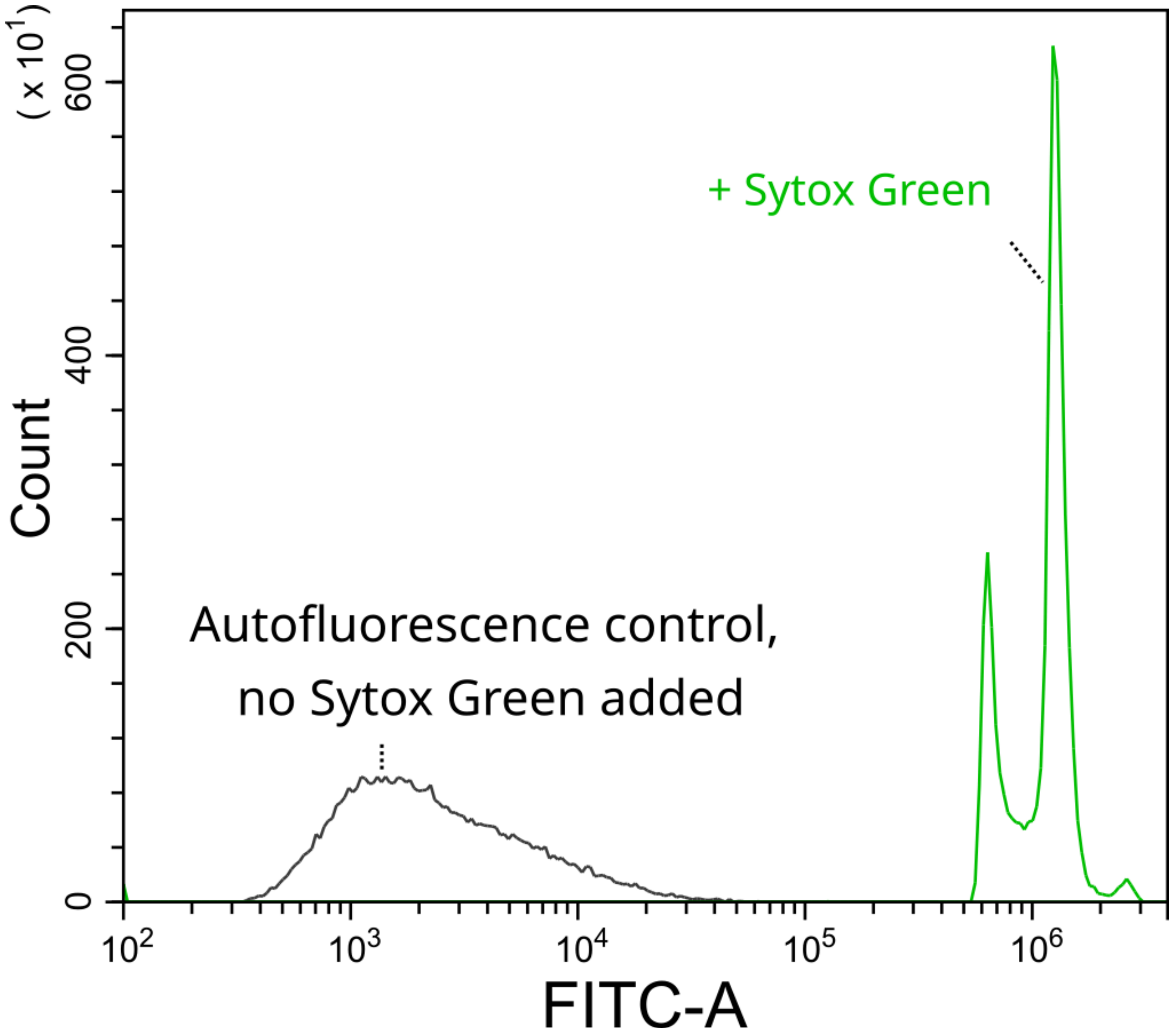

Figure S3. Comparison of autofluorescence and Sytox Green signal in yeast cells *S.cerevisiae*

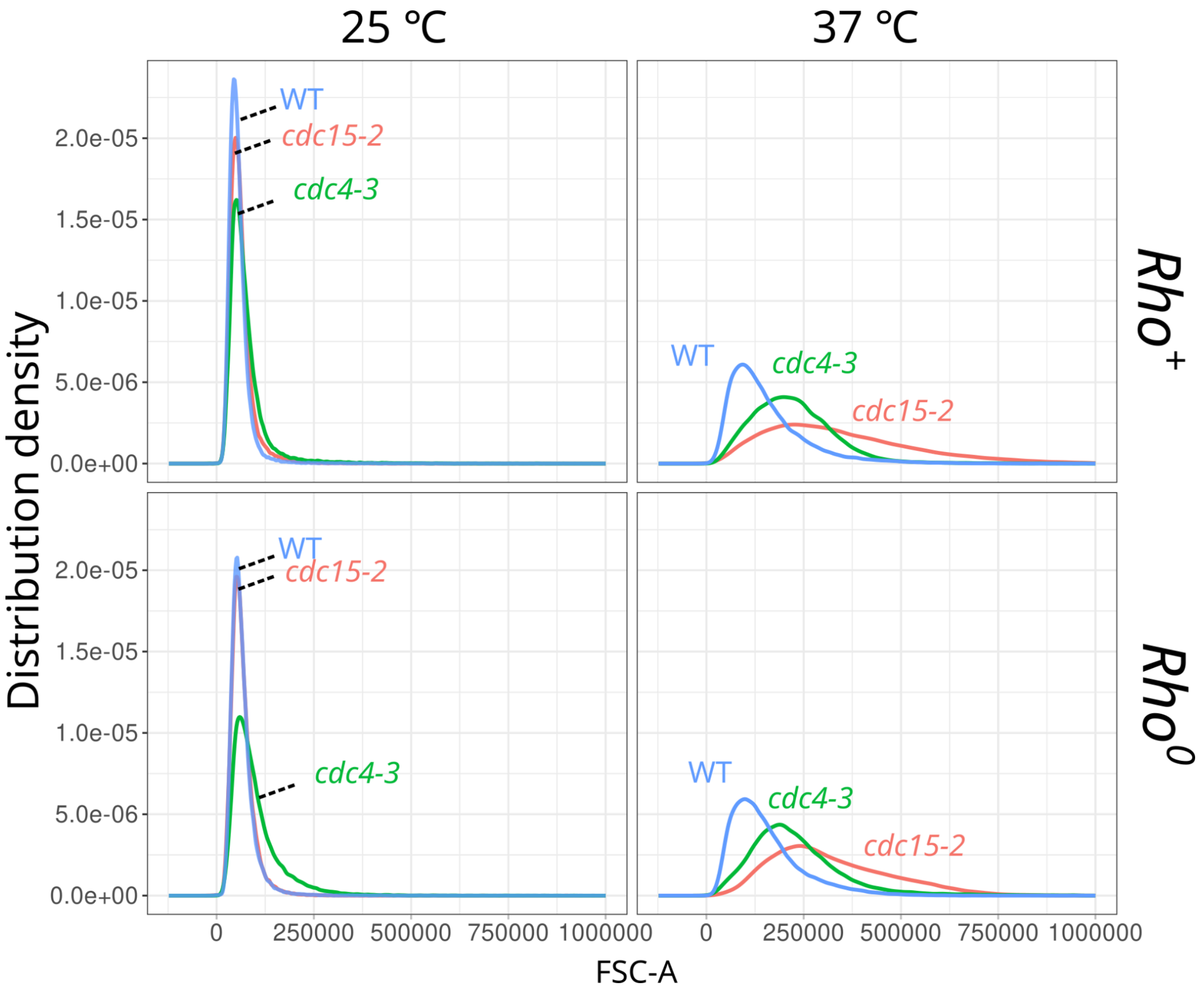

Figure S4. Cell size increase in yeast strains after temperature-induced cell cycle arrest. The distribution density of forward scatter area (FSC-A) parameters is shown for wild-type (WT), *cdc4-3*, and *cdc15-2* yeast strains. Cells were analysed before (25°C) or after six hours of incubation at non-permissive (37°C) temperature. Data correspond to the experiment presented in Figure 2

Calculation of mtDNA copy number in *rho*$^+$ cells

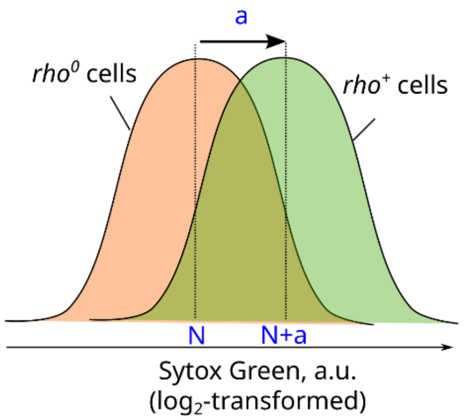

12.1 is the 1n nuclear genome size in kb
0.085 is the mitochondrial genome size in kb

a represents the surplus of Sytox Green signal in *rho*$^+$ cells on a $\log_2$-transformed scale

Sytox Green signal in *rho*$^+$ cells on a linear scale = $2^{N+a}-2^N = 2^N\times(2^a-1)$ where:

(2^a-1) represents the proportion of Sytox Green signals in *rho*$^+$ compared to *rho*^{0}

2^N ccorresponds to the Sytox Green signal from nuclear DNA (nDNA), therefore:

mtDNA total length = n × 12.1 kb × (2^a-1) *(Eq. 1)*
where n is nDNA ploidy (1 or 2)

b **= mtDNA copy number** = mtDNA total length /0.085 *(Eq. 2)*

Calculation of mtDNA copy number in *rho*$^+$ cells in cell-cycle arrested cells

c is the surplus of Sytox Green signal in *rho*$^+$ cell cycle arrested cells compared to *rho*$^+$ asynchronous cells in log2 scale

d is the surplus of Sytox Green signal in *rho*^{0} cell cycle arrested cells compared to *rho*^{0} asynchronous cells in log2 scale

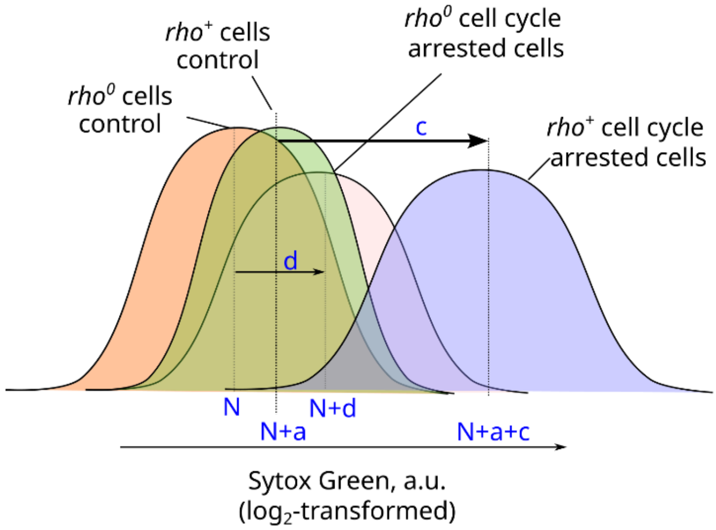

$2^{N+d}-2^N = 2^N\times(2^d-1)$ is the surplus of Sytox Green signal in *rho*^{0} cells on a linear scale compared to control *rho*^{0} non-arrested cells (autofluorescence)

mtDNA total length surplus in arrested cells =
$= (2^{N+a+c}-2^{N+a}) = 2^{N+a} \times (2^c-1)$
(Eq. 3)
is the surplus of Sytox Green signal in *rho*$^+$ cells on a linear scale in cell cycle arrested cells compared to control *rho*$^+$ non-arrested cells where 2^{N+a} corresponds to the Sytox Green signal in *rho*$^+$ cells
(12.1× n kb + b × 0.085) kb

mtDNA total length surplus in arrested cells (with correction of *rho*^{0} autofluorescence in arrested cells) =
$= 2^{N+a} \times (2^c-1) - 2^N \times (2^d-1) =$
$(12.1 + b \times 0.085)\ \text{kb} \times (2^c-1) - 12.1\ \text{kb} \times (2^d-1)$
(Eq. 4)

mtDNA copy number after cell cycle arrest =

$$= \frac{(12.1\times n + b \times 0.085)\ \text{kb} \times (2^c-1) - 12.1\ \text{kb} \times n \times (2^d-1)}{0.085\ \text{kb}} + 1$$

(Eq. 5)

Figure S5. Schematic representation and formulas illustrating the method for determining the mtDNA copy number in *rho*⁺ cells, as well as in cell-cycle arrested *rho*⁺ cells, based on the surplus of Sytox Green fluorescence signal. For calculations using the imputed experimental data, refer to Supplementary Table S2.

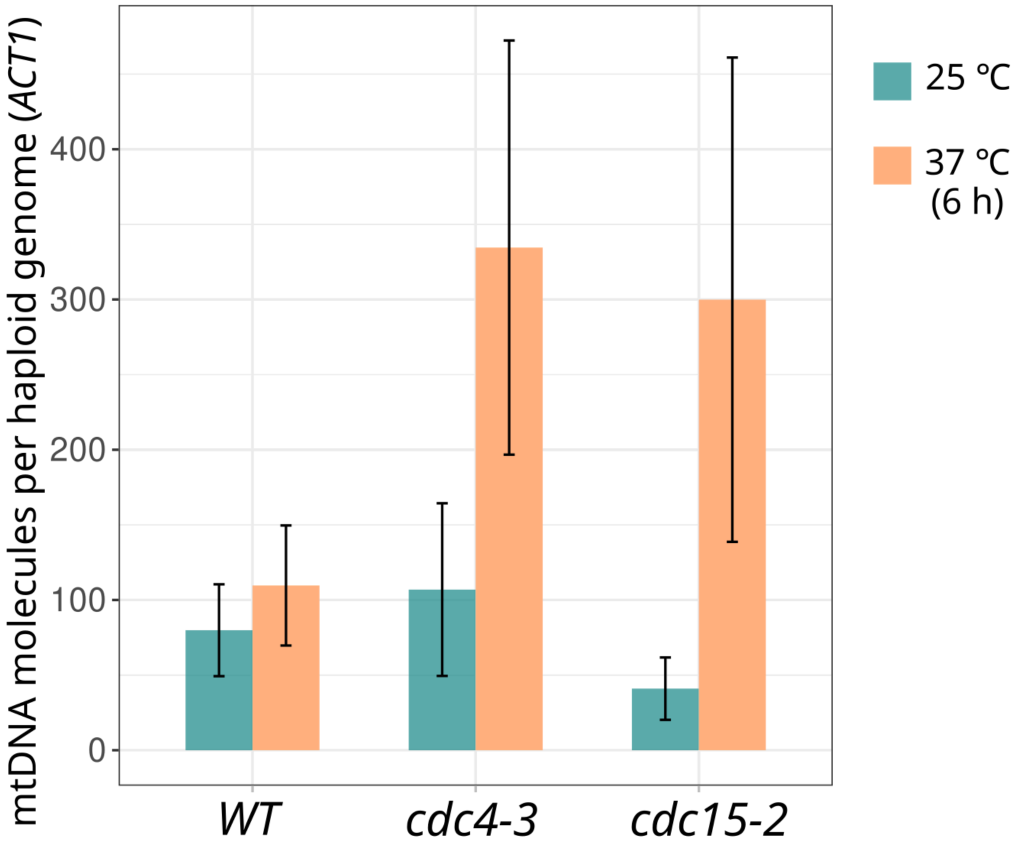

Figure S6. mtDNA copy number (Mito2 primer set) normalised to haploid nuclear genome (ACT1 gene) assessed using qPCR. Related to Figure 2E.

Table S1. Sequences of the qPCR primers

Target	Forward primer	Reverse primer
ACT1 (nDNA)	tcccaggtattgccgaaagaatgc	gccaagatagaaccaccaatccagac
Mito1 (mtDNA)	attccaccttcagcgtagt	ggttcggtcctcccttac
Mito2 (mtDNA)	ttcgcactaatcactcatcac	ccctacggtaactgtatttcaac
